# Analysis of profibrogenic microRNAs (miRNAs) expression in urine and serum of chronic kidney disease (CKD) stage 1–4 patients and their relationship with proteinuria and kidney function

**DOI:** 10.1007/s11255-021-02928-1

**Published:** 2021-07-27

**Authors:** Rafał Donderski, Joanna Szczepanek, Natalia Naruszewicz, Renata Naruszewicz, Andrzej Tretyn, Natalia Skoczylas-Makowska, Janusz Tyloch, Grażyna Odrowąż-Sypniewska, Jacek Manitius

**Affiliations:** 1 Department of Nephrology, Hypertension and Internal Medicine, University Hospital in Bydgoszcz Nicolaus Copernicus University, Toruń, Poland; 2 Skłodowskiej-Curie No 9, 85-094 Bydgoszcz, Poland; 3 Centre for Modern Interdisciplinary Technologies, Nicolaus Copernicus University, Toruń, Poland; 4 Department of Plant Physiology and Biotechnology, Nicolaus Copernicus University, Toruń, Poland; 5 Hemodialysis Unit, Davita Poland, Nakło, Poland; 6 Department of Biotechnology, Institute of General and Molecular Biology, Nicolaus Copernicus University, Toruń, Poland; 7 Department of Clinical Pathology, University Hospital in Bydgoszcz, Nicolaus Copernicus University, Toruń, Poland; 8 Department of Urology, University Hospital in Bydgoszcz, Nicolaus Copernicus University, Toruń, Poland; 9 Department of Clinical Laboratory Medicine, University Hospital in Bydgoszcz, Nicolaus Copernicus University, Toruń, Poland

**Keywords:** Chronic kidney disease (CKD), microRNAs expression, Tubulointerstitial compartment damage, Kidney fibrosis

## Abstract

**Purpose:**

Besides conventional kidney diseases diagnostics, micro RNAs (miRNAs) assessment in urine and serum is considered to be a promising non-invasive method of diagnostics of renal parenchymal diseases and valuable therapeutic target also. The purpose of the study was to investigate the role of several miRNAs as a markers of kidney damage.

**Methods:**

Assessment of 45 chronic kidney disease (CKD) patients stage 1–4 and 17 healthy control. Sample of urine and blood was taken from each participant for molecular analysis using Real Time PCR method to identify such micro-RNAs as: hsa-miR-155-5p, hsa-miR-214-3p, hsa-miR-200a-5p, hsa-miR-29a-5p, hsa-miR-21-5p, hsa-miR-93-5p, and hsa-miR-196a-5p. Basic biochemical test was done. Analysis was performed in CKD patients group and subgroup with chronic glomerulonephritis (CGN) confirmed by kidney biopsy. Moreover, analysis was performed in subgroup with different estimated glomerular filtration rate (eGFR) (according to CKD–EPI equation: eGFR < 60 ml/min, eGFR > 60 ml/min) and different daily protein excretion (DPE): (DPE < 3.5 g; DPE > 3.5 g).

**Results:**

Increased relative expression of hsa-miR-29-5p, hsa-miR-21-5p, and hsa-miR-196a-5p and decreased expression of hsa-miR-155-5p, hsa-miR-214-5p, hsa-miR-200a-5p, and hsa-miR-93-5p was demonstrated in urine of analyzed CKD patients. In subpopulation of chronic glomerulonephritis (CGN) patients, there was higher level of expression in urine of hsa-miR-155-5p, hsa-miR 214-3p, hsa-miR-93-5p, and hsa-miR-196a-5p in CGN with DPE < 3.5 g. CGN patients with eGFR < 60 ml/min showed higher expression level of miRNAs such as hsa-miR-214-3p, hsa-miR-29-5p, hsa-miR-93-5p, and hsa-miR-196-5p in urine. There was increase in hsa-miR 155-5p, hsa-miR-214-3p, and hsa-miR-200a-5p serum expression level in CKD population and reduction of hsa-miR-29a-5p, hsa-miR-21-5p, and hsa-miR-93-5p expression. Increased level of expression of hsa-miR-155-5p; hsa-miR-214-3p, hsa-miR-200a-5p, and hsa-miR-29-5p was found in CGN patients with eGFR > 60 ml/min.

**Conclusion:**

Increased relative expression of profibrogenic miRNAs in urine or serum of CKD patients with eGFR > 60 ml/min and DPE < 3.5 g may indicate higher degree of fibrosis at early CKD stages.

## Introduction

Chronic kidney disease (CKD) affects approximately 11% of adults worldwide and it seems to be an increasing health problem both in Western countries and low-income countries [1, 2]. Diabetes mellitus, hypertension, primary glomerulonephritis are three leading causes of CKD and end-stage kidney disease (ESKD). Irrespective of primary origin of renal damage the final common pathway is tubulointerstitial fibrosis which determine final outcome. Tubulointerstitial damage is initiated at early stages of CKD and it is a complex of pathological processes characterized by increased oxidative stress, inflammation, neoangiogenesis and fibrosis [3, 4]. Chemokines such as TGF-β, MCP-1, RANTES, PDGF, Ang II and others are involved. Percutaneous fine-needle kidney biopsy is considered to be a gold standard in the diagnostics of kidney diseases. Histological analysis of renal tissue may be helpful in detecting structural changes, both in glomerular and tubulointerstitial compartment and seems to be decisive in further strategy of nephroprotection. Disadvantages of kidney biopsy are an invasive nature of this procedure and possible complications. Therefore, we are still strong need for novel non-invasive methods of kidney diseases assessment. The novel approach in diagnostics of kidney disease comprise gene expression analysis in renal tissue, analysis of urinary proteins (especially low molecular weight protein) and miRNAs analysis. MiRNAs are small, non-coding RNA oligonucleotides that act as an important negative regulators of gene expression. They are important in both physiological processes but their dysregulation play also role in many pathological conditions. MiRNAs regulate cells proliferation, differentiation, inflammation, angiogenesis and oncogenesis [5]. They are associated with various diseases such as cancer (i.e. renal cell carcinoma), heart failure, coronary artery disease, kidney diseases, and allograft dysfunction [6].

MiRNAs are considered to be not only biomarkers but therapeutic target also. For example, mir208a has been identified as a potential biomarker of early phase of myocardial infarction [7]. The miR-21 is strongly related with pathogenesis of tubulointerstitial fibrosis in native kidney and in kidney allograft and higher expression of mir21 was reported in this condition [8]. On the other hand, endogenous miR-204-5p which is one of the most abundantly expressed miRNAs in human kidneys play a protective role in kidneys injury related with long-lasting hypertension and diabetic kidney disease (DKD) [9].

The aim of the study was to assess expression of microRNAs such as: hsa-miR-29-5p, hsa-miR-21-5p, hsa-miR-196a-5p, hsa-miR-155-5p, hsa-miR-214-5p, hsa-miR-200a-5p, and hsa-miR-93-5p in serum and in urine and establish relationship with kidney function filtration rate and proteinuria. Additional analyses were performed in subpopulation of chronic glomerulonephritis (CGN) patients.

## Materials and methods

### Study population

The study included 45 Caucasian patients. There were: 21F and 24 M (mean age 52.36 ± 15.90). The patients were recruited from Outpatient Clinic of Department of Nephrology, Hypertension and Internal Medicine at Ludwik Rydygier University Hospital in Bydgoszcz. The control group consisted of 17 healthy volunteers matched according to age and sex (mean age 50.59 ± 8.75). Written informed consent was obtained from all participants included in the study. Fasting blood samples (of 2–3 ml volume) and samples of morning urine were collected for laboratory analyses.

The diagnosis of CKD was based on KDIGO 2012 Guidelines. The causes of CKD were: diabetic kidney disease—3 patients, biopsy proven chronic glomerulonephritis (CGN)—21 patients; tubulointerstitial nephropathy (including urolithiasis, obstructive nephropathy, gout nephropathy)—8 patients; polycystic kidney disease—6 patients; hypertensive nephropathy—6 patients; unknown origin: 1 patient. There were 8 patients at stage 1 CKD; 9 patients at stage 2; 17 patients CKD stage 3; 11 patients at stage 4 CKD. Inclusion criteria were: age > 18 years old; CKD stage 1–4; stable clinical condition 4 weeks prior to enrollment (no signs of any acute illness), no signs of inflammation (CRP < 5.0 mg/l) or active immunological disease with renal involvement, written informed consent. Exclusion criteria were: active immunosuppressive therapy, CKD stage 5. Comorbid conditions were: coronary artery disease (CAD) diagnosed in 6 patients, hypertension 44 patients, symptoms of congestive heart failure (CHF) were present in 2 patients; The patients who suffered from hypertension, required 2 or more antihypertensive agents. Treatment of hypertension was in accordance with current guidelines of ESH/ESC (2018). The goal of treatment was to reach blood pressure 130-139/70-79 mmHg if tolerated. The following medications were prescribed: angiotensin converting enzyme inhibitors (ACE-Is)—21 patients, angiotensin receptor blockers (ARBs)—18 patients, calcium channel blockers—24; β-blockers—24 patients, loop diuretics—24 patients. Statins were prescribed in 25 patients. eGFR was calculated in each participant according to CKD-EPI formula. Renal anemia was treated with darbepoetin-alfa use subcutaneously according to KDIGO Clinical Practice Guidelines to reach hemoglobin level between 10.0 and 11.5 g/dl. The drug was prescribed in 2 patients. Chronic kidney disease—mineral and bone disorder (CKD-MBD) were treated according to KDIGO guidelines 2016 (Uptade on the management of CKD-MBD) using conventional therapy: vitamin D (paricalcitol in a dose 1.0 mcg daily 3 times a week) and calcium–phosphorus binders (calcium carbonate in a dose from 3.0 to 6.0 g daily). Dietary counseling was performed in each CKD individuals by dietician. In all patients and controls clinical evaluation with measurements of body mass, height, and BMI calculations were performed. The clinical characteristics of the patients is presented in Table 1. The study protocol was approved by the local Bioethics Committee of the Nicolaus Copernicus University Collegium Medicum in Bydgoszcz (Reference number: 114/2018).

**Table 1:** Clinical characteristics and baseline biochemical parameters of CKD patients and control group

Parameter	CKD group (*n* = 45)	Control group (*n* = 17)	*p*
Age (years)	52.36 ± 15.90	50.59 ± 8.75	0.7776
BMI (kg/m^2^)	27.65 ± 5.13	24.28 ± 3.30	0.0039
Serum creatinine (mg/dl)	1.58 ± 0.84	0.83 ± 0.17	< 0.0001
eGFR (CKD-EPI) ml/min/1.73 m^2^	56.47 ± 27.01	92.06 ± 17.11	< 0.0001
Hb (g/dl)	14.01 ± 1.66	14.41 ± 0.89	0.2221
Alb (g/dl)	4.29 ± 0.42	4.43 ± 0.28	0.1458
TP (g/dl)	7.02 ± 0.45	6.82 ± 0.31	0.1121
TCH (mg/dl)	202.14 ± 52.09	200.5 ± 43.6	0.8141
LDL-C (mg/dl)	114.74 ± 39.0	127.6 ± 27.22	0.1747
HDL-C (mg/dl)	51.7 (46.00–59.15)	61.5 (55.90–79.95)	< 0.05
TG (mg/dl)	142.8 (98.75–187.70)	103.6 (87.00–139.85)	0.24
CRP (mg/l)	1.5 (0.59–3.55)	0.8 (0.32–1.25)	0.05
Fibrinogen (mg/dl)	346.9 ± 99.41	240.41 ± 36.21	< 0.0001
UA (mg/dl)	6.89 ± 1.88	5.16 ± 1.05	< 0.0001
Calcium (mg/dl)	9.29 ± 0.41	9.0 ± 0.49	0.0377
Phosphorus (mg/dl)	3.58 ± 0.56	3.45 ± 0.55	0.8853

Normally distributed data are expressed as mean ± SD and not normally distributed data are expressed as median (IQR)

*BMI* body mass index, *eGFR* estimated glomerular filtration rate according to CKD-EPI equation, *Hb* hemoglobin, *Alb* serum albumin, *TP* serum total protein, *TCH* total cholesterol, *LDL-C* low density lipoprotein cholesterol, *HDL-C* high density lipoprotein cholesterol, *CRP* C-reactive protein, *TG* triglycerides, *UA* uric acid

Histopathological characteristics of chronic glomerulonephritis (CGN) patients is presented in Table 2.

**Table 2:** Histopathological characteristics of chronic glomerulonephritis (CGN) patients

Histopathology evaluation	Number of cases
MCD	2
IgA nephropathy	11
FSGS	2
Membranous nephropathy	2
Mesangioproliferative glomerulonephritis	2
MPGN	2

*MCD* minimal change disease, *FSGS* focal segmental glomerulosclerosis, *MPGN* membranoproliferative glomerulonephritis

### Molecular analysis

The 7 selected miRNAs were analyzed using quantitative real-time PCR (qPCR) in a group of patients with CKD and controls.

#### Sample collection and microRNA extraction

The whole blood sample (~ 5 mL) from the patients was collected in EDTA-containing vacutainers and transferred into a microtube. The samples were centrifuged at 1000×*g* at 4 °C for 10 min (× 2). Plasma was collected carefully and aliquoted in RNase-free tubes and stored at − 80 °C until future use. All plasma samples were completely thawed on ice and centrifuged at 3000×*g* at 4 °C for 5 min in to remove the remaining cell debris.MiRNAs from 200 µl of serum were isolated using the miRCURY™ RNA Isolation Kit-Biofluids (EXIQON). Urine samples (~ 2 mL) were centrifuged at 4 °C at 10,000×*g*. MiRNAs from 200 µl of urine samples were extracted using MagMAX mirVana Total RNA Isolation Kit (Applied Biosystem™ by TermoFisher Scientific). Exosomes were isolated from urine using the Total Exosome Isolation Reagent (from urine) (Invitrogen™ by Thermo Fisher Scientific). All extractions and preparation of the miRNA samples were conducted according to the manufacturer’s instructions. RNA Spike-In and SP2 were applied as internal controls. The Nanodrop ND-2000 (Thermo Scientific) was used for the quantification of samples.

#### Reverse transcription (RT) reaction

Complementary DNA was synthesized using TaqMan^®^ Advanced miRNA cDNA Synthesis Kit (Applied Biosystem™ by Life TechnologiesTM) with Universal RT primers. This cDNA synthesis kit uses 3ʹ poly-A tailing and 5ʹ ligation of an adaptor sequence to extend the mature miRNAs present in the sample on each end. To improve detection of miRNA, the obtained cDNA were amplified using the Universal miR-Amp Primers and miR-Amp Master Mix to uniformly increase the amount of cDNA for each target. According to the manufacturer’s recommendations, 3 steps were performed:

1. the poly(A) tailing reaction (mix containing 2 μL of miRNA, 0.5 μL 10X Poly(A) Buffer, 0.5 μL ATP 0.3 μL Poly(A) Enzyme and 1.7 μL RNase-free water were prepared, and were incubated at 37 °C for 45 min, and 65 °C for 10 min in a thermalcycler);
2. the adaptor ligation reaction (to each reaction tube containing the adaptor ligation reaction product was transfer 15 μL of the RT Reaction Mix containing 6 μL 5X RT Buffer, 1.25 ul dNTP Mix (25 mM each), 1.5 μL 20X Universal RT Primer, 3 μL 10X RT Enzyme Mix and 3.3 ul RNase-free water and was incubated using the following cycling 15 min at 42 °C, and 5 min at 85 °C); and
3. the miR-Amp reaction (5 μL of the miR-Amp Reaction Mix (25 μL 2X miR-Amp Master Mix and 2.5 μL 20X miR-Amp Primer Mix, completed with RNase-free water) were transferred to 5 μL of the RT reaction product and samples were incubated at 95 °C for 5 min, followed by 14 cycles at 95 °C for 3 s and 60 °C for 30 s).

#### Real-time PCR

7 miRNAs (hsa-mir-155-5p, hsa-mir-214-3p, hsa-mir-200a-5p, hsa-mir-29a-5p, hsa-mir-21-5p, hsa-mir-93-5p, and hsa-mir-196a-5p) were selected for analysis based on involvement in pathways relevant to CKD, especially renal fibrosis according to relevant literature and bioinformatics tools (GeneCards^®^—the Human Gene Database TargetScan, miRDB—MicroRNA Target Prediction Database, and HMDD—the Human microRNA Disease Database). The expression level of miRS was examined by TaqMan^®^ Advanced miRNA Assays (Applied Biosystems).

**Table:** 

miRBase ID	Chromosome location	Mature miRNA sequence	Targets (miRDB)
hsa-mir-155-5p	Chr.21: 25,573,980–25,574,044 [+]	UUAAUGCUAAUCGUGAUAGGGGUU	701 predicted targets
hsa-mir-214-3p	Chr.1: 172,138,798–172,138,907 [−]	ACAGCAGGCACAGACAGGCAGU	322 predicted targets
hsa-mir-200a-5p	Chr.1–1,167,863–1,167,952 [+]	UAACACUGUCUGGUAACGAUGU	438 predicted targets
hsa-mir-29a-5p	Chr.7–130,876,747–130,876,810 [−]	ACUGAUUUCUUUUGGUGUUCAG	885 predicted targets
hsa-mir-21-5p	Chr.17: 59,841,266–59,841,337 [+]	UAGCUUAUCAGACUGAUGUUGA	469 predicted targets
hsa-mir-93-5p	Chr.7: 100,093,768–100,093,847 [−]	CAAAGUGCUGUUCGUGCAGGUAG	1319 predicted targets
hsa-mir-196a-5p	Chr17: 48,632,490–48,632,559 [−]	UAGGUAGUUUCAUGUUGUUGG	370 predicted targets

Real-time PCR amplifications were performed using TaqMan MicroRNA Assays, consisting of an RNA-specific stem-looped RT primer and TaqMan^®^ Assay (forward and reverse primers and FAM™ dye-labeled MGB probe. The 20 μL reactions mixes were prepared. Each qPCR was performed in duplicate and contained 5 μL of 10× diluted reverse transcription product, 10 μL TaqMan^®^ Fast Advanced Master Mix (2X) and 1 μL of primer and hydrolysis probe mix of the TaqMan^®^ Advanced miRNA Assay (20X). After 20 s incubation at 95 °C, amplification was performed for 40 cycles at 95 °C for 1 s and at 60 °C for 20 s. Each sample was run in duplicate in each qPCR reaction. The amplification reaction was performed on the LightCycler^®^ 480 (Roche, Basel, Switzerland). The cycle number at which the amplification plot crossed the threshold was calculated (CT). Expression levels of mature miRNAs were calculated based on the comparative threshold cycle method (RQ = 2^−ΔΔC*t*^). Relative expression levels were calculated using miRNA levels after normalization. Threshold cycle (C*t*) values of selected miRs were normalized to hsa-mir-484 and hsa-mir-16.

### Biochemical evaluation

The blood sample were taken in each subjects for assessment of serum creatinine, serum albumin (Alb), total protein (TP), uric acid (UA), C-reactive protein (CRP), hemoglobin (Hb), total cholesterol (TCH), LDL-cholesterol (LDL-C), HDL-cholesterol (HDL-C), triglycerides (TG), serum calcium (Ca), serum phosphorus (P), fibrinogen (g/dl) were measured by routine laboratory methods.

### Statistical analysis

Statistical analysis was performed using the Statistica 7.0 PL software (StatSoft Inc., Tulsa, OK, USA). The obtained data are presented as mean ± standard deviation (SD) and the median and top and bottom quartiles are given for variables that were not normally distributed. Distribution of variables was analyzed using the Shapiro–Wilk test. Statistical analysis was performed using the Student’s *t* test. If any variable was not normally distributed, the* U *Mann–Whitney test was used. Qualitative data were compared by means of the *χ*^2^ test. Linear correlation between variables was analyzed using: Spearman rank correlation coefficient (for samples with non-normal distribution) and Pearson’s correlation coefficient (for samples with normal distribution) also. *p* value < 0.05 was considered as statistically significant.

## Results

There was an increase of the relative expression level of three miRNAs molecules: hsa-miR-29-5p, hsa-miR-21-5p, and hsa-miR-196a-5p in urine of CKD patients. The hsa-miR-29-5p molecule showed the highest, almost twofold increase in expression. The expression of hsa-miR-21-5p and hsa-miR-196a-5p in the study group increased 1.15-fold and 1.26-fold, respectively, compared to the control group. The other molecules: hsa-miR-155-5p, hsa-miR-214-5p, hsa-miR-200a-5p, and hsa-miR-93-5p are characterized by a reduced level of expression in the urine in the CKD group. It was shown an over sevenfold decrease in expression of mir-155-5p, 1.3-fold, 3.2-fold, and 1.7-fold reduction in expression, of hsa-miR-214-5p, hsa-miR-200a-5p and hsa-miR-93-5p respectively, compared to the control group. These data are presented in Table 3. Moreover, increased expression of the same miRNAs i.e. hsa-miR-29-5p, hsa-miR-21-5p, and hsa-miR-196a-5p was detected in subgroup of CGN and non-CGN and in further analysis of CKD groups with different filtration function (i.e. eGFR < 60 ml/min and eGFR > 60 ml/min) and proteinuria (i.e. DPE < 3.5 g and DPE > 3.5 g) (Table 3). We also analyzed the serum and urine level of abovementioned miRNAs expression in subpopulation of CGN patients. In urine, there was higher relative level of expression of hsa-miR-155-5p, hsa-miR 214-3p, hsa-miR 93-5p, and hsa-miR 196a-5p in CGN with DPE below 3.5 g (Table 3). Interestingly, in CGN pts with eGFR < 60 ml/min there was higher expression level of 4 miRNAs such as hsa-miR-214-3p, hsa-miR-29-5p, hsa-miR-93-5p, hsa-miR-196-5p (Table 3).

**Table 3:** The relative expression of miRNAs: hsa-miR-29-5p, hsa-miR-21-5p, hsa-miR-196a-5p, hsa-miR-155-5p, hsa-miR-214-5p, hsa-miR-200a-5p and hsa-miR-93-5p in urine of CKD, CGN and non-CGN patients with different renal function (eGFR > 60 ml/min/1.73m^2^ and eGFR < 60 ml/min/1.73m^2^) and different proteinuria (DPE > 3.5 g and DPE < 3.5 g)

	hsa-mir-155-5p	hsa-mir-214-3p	hsa-mir-200a-5p	hsa-mir-29-5p	hsa-mir-21-5p	hsa-mir-93-5p	hsa-mir-196a-5p
R CKD vs control	0.14	0.73	0.32	1.88	1.15	0.59	1.26
R CGN vs control	0.18	0.82	0.11	1.56	1.00	0.73	1.32
R Non-CGN vs control	0.11	0.68	0.60	2.06	1.25	0.52	1.22
R DPE < 3.5 g vs control	0.16	0.78	0.38	1.91	1.05	0.70	1.30
R DPE > 3.5 g vs control	0.06	0.37	0.04	1.45	1.15	0.60	1.26
R CKD-EPI < 60 ml/min vs Control	0.11	0.80	0.36	1.96	1.30	0.57	0.83
R CKD-EPI > 60 ml/min vs control	0.19	0.64	0.26	1.74	0.98	0.63	1.01

Subpopulation of CGN	hsa-mir-155-5p	hsa-mir-214-3p	hsa-mir-200a-5p	hsa-mir-29-5p	hsa-mir-21-5p	hsa-mir-93-5p	hsa-mir-196a-5p
R DPE > 3.5 g vs control	0.12	0.42	0.06	No expression	0.93	0.58	0.23
R DPE < 3.5 g—control	3.33	1.41	0.24	0.76	0.78	1.64	1.38
R CKD-EPI > 60 ml/min vs control	2.13	0.81	0.13	0.37	0.76	1.19	0.84
R CKD-EPI < 60 ml/min vs control	0.70	3.45	0.53	2.66	0.92	2.17	2.12

Additional analysis was also performed in subpopulation of CGN

*R *relative expression of MiRNAs, *CKD* chronic kidney disease, *CGN* chronic glomerulonephritis, *DPE* daily protein excretion

In the serum of analyzed CKD patients, it comes to increase in hsa-miR-155-5p; hsa-miR-214-3p, and hsa-miR-200a-5p expression levels. On the other hand, hsa-miR-29a-5p, hsa-miR-21-5p, and hsa-miR-93-5p showed lower expression in patients in the CKD group in compare to the control group. These data are presented in Table 4. We noticed even threefold increase of mir-200-5p expression. There was 2, fivefold lower expression of hsa-miR-29a-5p and an almost twofold decrease in expression level of hsa-miR-21-5p in CKD group. We also reported twofold lower level of hsa-miR-93-5p expression in serum. Small changes of expression level was confirmed for hsa-miR-196a-5p (Table 4). Assessment of relative expression of selected miRNAs in CKD population with different eGFR and DPE was also done. (Table 4). Moreover, it is worth to mention that in subanalysis of CGN pts we found increased expression of hsa-miR-155-5p; hsa-miR 214-3p, hsa-miR 200a-5p and hsa-miR-29-5p in patients with eGFR > 60 ml/min. Extremely high expression level was also detected for hsa-miR-155-5p; hsa-miR-214-3p; hsa-miR-200a-5p; hsa-miR-29-5p and hsa-miR-196-5p in CGN patients with nephrotic syndrome range proteinuria (Table 4). Linear correlation between ΔCt values for analyzed profibrogenic miRNAs and some biochemical and clinical parameters such as serum creatinine concentration, eGFR, hemoglobin, albumin, total calcium, phosphorous, total cholesterol, daily protein excretion, systolic and diastolic blood pressure (SBP, DBP) are presented in Tables 5 and 6. The results for multiple linear regression analysis for selected miRNAs as a dependent variable in patients with CKD1-4 are given in Tables 7 and 8.

**Table 4:** The relative expression of hsa-miR-29-5p, hsa-miR-21-5p, hsa-miR-196a-5p, hsa-miR-155-5p, hsa-miR-214-5p, hsa-miR-200a-5p and hsa-miR-93-5p in serum of CKD patients, CGN and non-CGN patients with different renal function (eGFR > 60 ml/min/1.73 m^2^ and eGFR < 60 ml/min/1.73 m^2^) and different proteinuria (DPE > 3.5 g and DPE < 3.5 g)

	hsa-mir-155-5p	hsa-mir-214-3p	hsa-mir-200a-5p	hsa-mir-29-5p	hsa-mir-21-5p	hsa-mir-93-5p	hsa-mir-196a-5p
R CKD vs control	1.06	1.40	2.69	0.41	0.51	0.48	0.96
R CGN vs control	0.80	0.94	2.70	0.74	0.36	0.45	0.85
R Non-CGN vs control	1.16	1.51	2.69	0.32	0.61	0.49	1.03
R DPE < 3.5 g vs control	0.89	1.10	2.45	0.32	0.46	0.43	0.81
R DPE > 3.5 g vs control	2.51	4.27	6015	5.00	1.22	.07	3.21
R CKD-EPI < 60 ml/min vs control	0.77	2.78	1.95	0.19	0.38	0.29	0.74
R CKD-EPI > 60 ml/min vs control	2.51	4.27	6.15	5.00	1.22	1.07	3.21

Subpopulation of CGN	hsa-mir-155-5p	hsa-mir-214-3p	hsa-mir-200a-5p	hsa-mir-29-5p	hsa-mir-21-5p	hsa-mir-93-5p	hsa-mir-196a-5p
R DPE > 3.5 g vs control	2.09	4.78	7.81	10.68	0.71	1.15	2.88
R DPE < 3.5 g vs control	0.64	0.66	2.26	0.55	0.31	0.36	0.65
R CKD-EPI > 60 ml/min vs control	1.40	1.53	4.32	1.35	0.58	0.70	1.09
R CKD-EPI < 60 ml/min vs control	0.15	0.18	0.49	0.30	0.08	0.10	0.18

Additional analysis was also performed in subpopulation of CGN

*R* relative expression of MiRNAs, *CKD* chronic kidney disease, *CGN* chronic glomerulonephritis, *DPE* daily protein excretion

**Table 5:** Summary of statistically significant linear correlation between miRNAs in patients with CKD and laboratory measurements

	hsa-mir-155-5p	hsa-mir-214-3p	hsa-mir-200a-5p	hsa-mir-29-5p	hsa-mir-21-5p	hsa-mir-93-5p	hsa-mir-196a-5p		hsa-mir-155-5p	hsa-mir-214-3p	hsa-mir-200a-5p	hsa-mir-29-5p	hsa-mir-21-5p	hsa-mir-93-5p
	*r*	*p*	*r*	*p*	*r*	*p*	*r*	*p*	*r*	*p*	*r*	*p*	*r*	*p*
eGFR(ml/min)	− 0.03	0.82	− 0.01	0.9	− 0.08	0.61	− 0.03	0.84	− 0.04	0.79	0.36	0.02	− 0.01	0.93
Creatinine (mg/dl)	0.05	0.74	0.11	0.47	0.15	0.36	0.11	0.51	0.11	0.45	**− 0.32**	**0.03**	0.07	0.63
Hb (g/dl)	− 0.21	0.17	− 0.03	0.81	− 0.20	0.22	− 0.14	0.44	− 0.12	0.43	**− 0.28**	**0.05**	− 0.11	0.48
Albumin (g/dl)	0.15	0.37	**0.32**	**0.04**	0.23	0.18	**− 0.38**	**0.03**	0.23	0.14	0.13	0.42	0.26	0.09
Calcium (mmo/l)	0.10	0.5	0.09	0.5	0.13	0.46	0.16	0.38	0.26	0.10	**0.32**	**0.04**	0.21	0.20

Results for serum assessment

*eGFR* estimated glomerular filtration rate, *Hb* hemoglobin concentration

**Table 6:** Summary of statistically significant linear correlation between miRNAs in patients with CKD patients and laboratory/clinical measurements

	hsa-mir-155-5p		hsa-mir-214-3p		hsa-mir-200a-5p		hsa-mir-29-5p		hsa-mir-21-5p		hsa-mir-93-5p		hsa-mir-196a-5p	
	*r*	*p*	*r*	*p*	*r*	*p*	*r*	*p*	*r*	*p*	*r*	*p*	*r*	*p*
eGFR (ml/min)	0.14	0.57	0.16	0.28	0.21	0.17	0.18	0.34	0.17	0.26	0.05	0.74	**− 0.31**	**0.03**
Creatinine (mg/dl)	− 0.22	0.35	− 0.15	0.32	− 0.26	0.09	− 0.22	0.24	− 0.20	0.18	0.10	0.53	**− 0.33**	**0.02**
Hb (g/dl)	**0.56**	**0.01**	0.19	0.21	− 0.14	0.36	− 0.03	0.88	− 0.23	0.11	− 0.07	0.65	0.23	0.13
Calcium (mmo/l/l)	− 0.31	0.23	− 0.27	0.09	− 0.16	0.33	**− 0.38**	**0.05**	0.10	0.55	0.12	0.48	− 0.18	0.26
SBP office	**0.45**	**0.05**	0.02	0.89	− 0.05	0.75	0.00	0.99	− 0.02	0.90	0.05	0.74	0.12	0.43
DBP office	0.14	0.55	0.13	0.38	− 0.09	0.54	− 0.04	0.83	− 0.03	0.82	0.10	0.53	0.21	0.15

Results for urine assessment

*eGFR* estimated glomerular filtration rate, *SBP* systolic blood pressure, *DBP* diastolic blood pressure, *Hb* hemoglobin concentration

**Table 7:** Results of multiple linear regression analysis in patients with CKD

MicroRNAs	Variable	Beta	Standard error Beta	*p*
mir-155-5p	Calcium	0.42	0.13	0.0035
	Hemoglobin	− 0.41	0.13	0.0042
	Fibrinogen	− 0.59	0.15	0.0006
	UA	0.64	0.16	0.0004
	eGFR	0.32	0.15	0.0419
	Multiple *r* = 0.66 *r*^2^ = 0.43 *p* = 0.0006			
mir-214-3p	DBP	− 0.53	0.11	< 0.0001
	Calcium	0.42	0.11	0.0004
	Fibrinogen	− 0.61	0.13	< 0.0001
	UA	0.57	0.13	< 0.0001
	Multiple *r* = 0.76 *r*^2^ = 0.58 *p* < 0.0001			
mir-200a-5p	Calcium	0.31	0.14	0.0381
	UA	0.36	0.17	0.0449
	Fibrinogen	− 0.35	0.17	0.0492
	Multiple *r* = 0.44 *r*^2^ = 0.19 *p* = 0.0374			
mir-29-5p	DBP	− 0.44	0.13	0.0019
	Calcium	0.37	0.13	0.0071
	Fibrinogen	− 0.46	0.16	0.0067
	UA	0.37	0.16	0.0225
	Multiple *r* = 0.61 *r*^2^ = 0.37 *p* = 0.0013			
mir-21-5p	Calcium	0.56	0.11	< 0.0001
	DBP	− 0.54	0.11	< 0.0001
	UA	0.41	0.13	0.0028
	Fibrinogen	− 0.39	0.13	0.0055
	Multiple *r* = 0.76 *r*^2^ = 0.57 *p* < 0.0001			
mir-93-5p	Calcium	0.51	0.10	< 0.0001
	DBP	− 0.54	0.10	< 0.0001
	Hemoglobin	− 0.42	0.10	0.0002
	UA	0.45	0.12	0.0004
	Fibrinogen	− 0.48	0.12	0.0004
	Multiple *r* = 0.82 *r*^2^ = 0.67 *p* < 0.0001			
mir-196a-5p	Calcium	0.52	0.10	< 0.0001
	DBP	− 0.54	0.11	< 0.0001
	Fibrinogen	− 0.60	0.13	< 0.0001
	UA	0.42	0.12	0.0019
	Hemoglobin	− 0.30	0.11	0.0084
	Multiple *r* = 0.79 *r*^2^ = 0.62 *p* < 0.0001			

Results for serum assessment

**Table 8:** Results of multiple linear regression analysis in patients with CKD

MicroRNAs	Variable	Beta	Standard error Beta	*p*
mir-155-5p	Hemoglobin	0.53	0.12	< 0.0001
	Total protein	− 0.46	0.12	< 0.0001
	BMI	− 0.32	0.12	0.0098
	SBP	0.31	0.12	0.0133
	Multiple *r* = 0.69 *r*^2^ = 0.48 *p* < 0.0001			
mir-214-3p	BMI	− 0.42	0.16	0.0114
	UA	0.42	0.17	0.0175
	Creatinine	− 0.35	0.15	0.0233
	Multiple *r* = 0.48 *r*^2^ = 0.23 *p* = 0.0137			
mir-200a-5p	Total cholesterol	0.41	0.13	0.0032
	UA	0.60	0.16	0.0006
	Creatinine	− 0.41	0.14	0.0048
	BMI	− 0.36	0.15	0.0203
	Multiple *r* = 0.62 *r*^2^ = 0.38 *p* = 0.0007			
mir-29-5p	No model			
mir-21-5p	No model			
mir-93-5p	No model			
mir-196a-5p	Creatinine	− 0.55	0.13	0.0002
	DBP	0.35	0.13	0.0108
	Multiple *r* = 0.57 *r*^2^ = 0.33 *p* = 0.0003			

Results for urine assessment

**Fig. 1:**
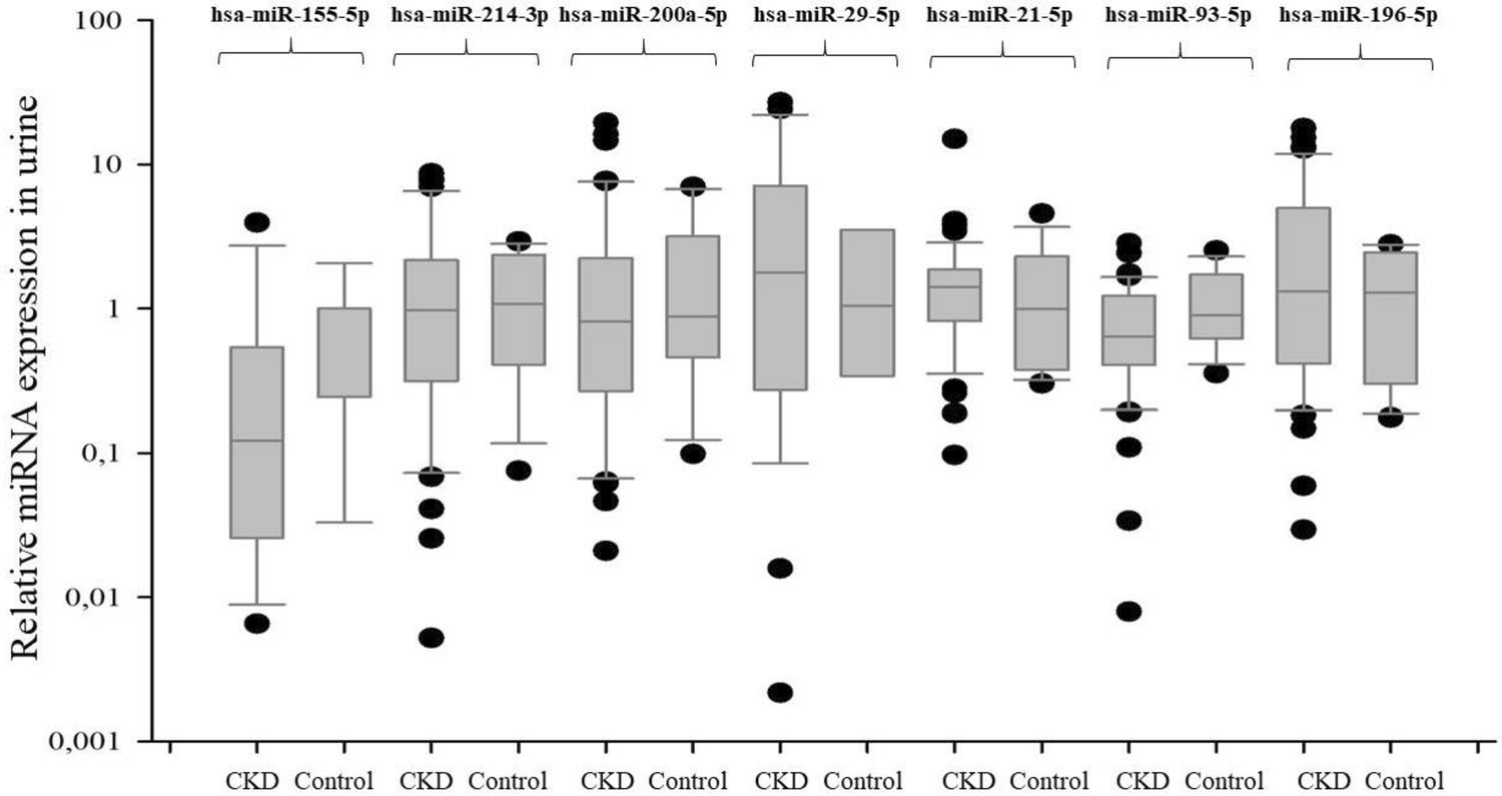
The relative expression of profibrogenic miRNAs in CKD patients (urine assessment)

**Fig. 2:**
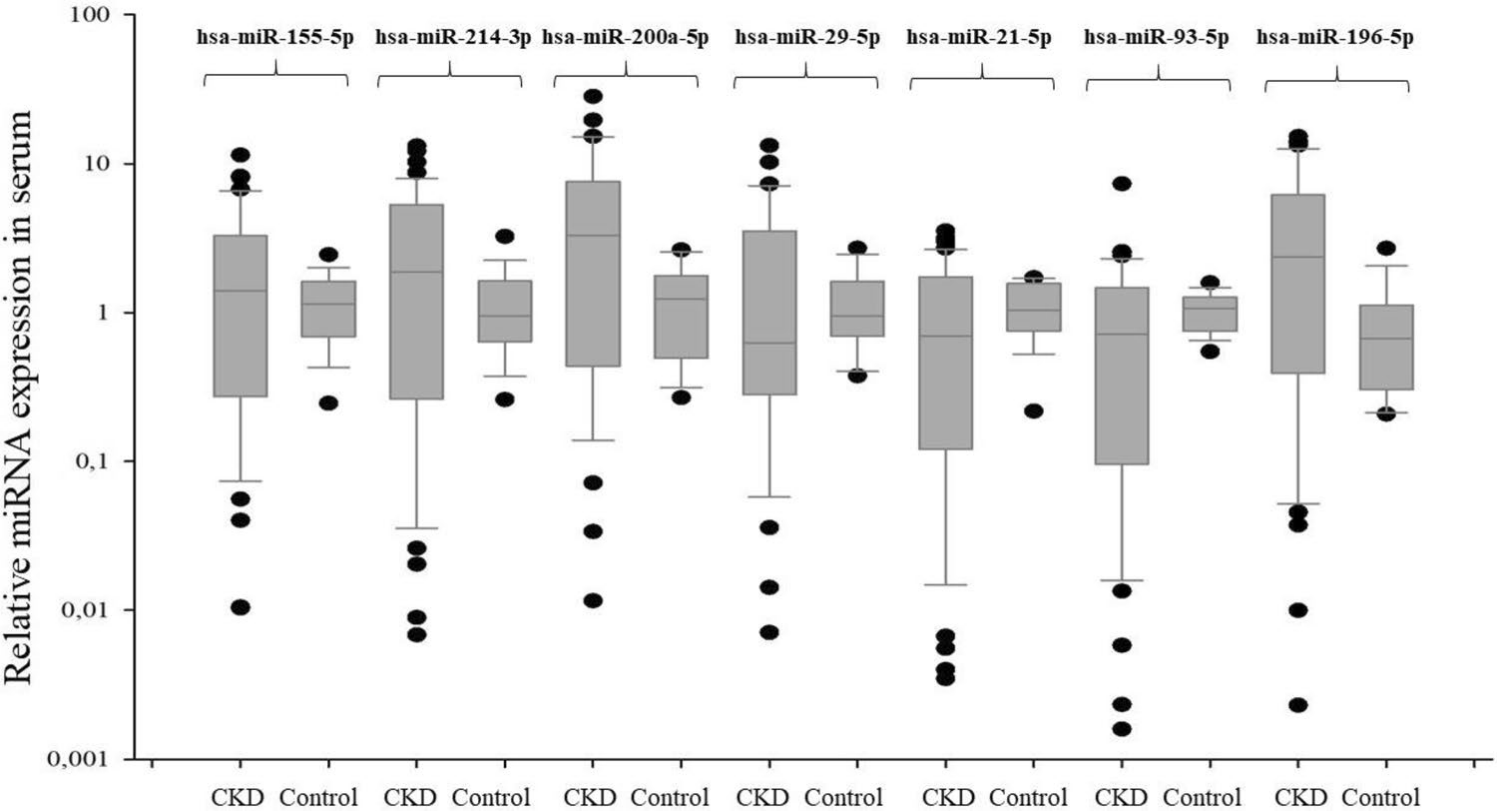
The relative expression of profibrogenic miRNAs in CKD patients (serum assessment)

For example, in multivariate analysis, the relative expression of hsa-mir 155-5p is significantly associated with serum calcium (*p* = 0.0035), hemoglobin concentration (*p* = 0.0042), fibrinogen (*p* = 0.0006), UA (*p* = 0.0004) and eGFR (*p* = 0.04). Hsa-mir-21-5p in serum is significantly associated with serum calcium (*p* < 0.0001), DBP (*p* < 0.0001), UA (*p* = 0.0028), fibrinogen (*p* = 0.0055). The relative expression of profibrogenic mi-RNAs in urine/serum of analyzed CKD population was shown in Figs. 1 and 2.

## Discussion

The miRNAs are playing important role in kidney physiology and pathology. Their role was proved in maintaining renal control over sodium and potassium handling and there is increasing evidence of their role in renal fibrosis. In literature, there are conflicting reports about changes of relative expression of profibrogenic miRNAs in urine, serum, and kidney tissue. Despite of this ambiguous reports, there have been a consent that miRNAs may participate in processes of inflammation, oxidative stress and regulation of TGF-β activity which are crucial in initiation of tubulointerstitial fibrosis. On the other hand some miRNAs (such as miR-200a-5p) have been shown to halt progression of fibrosis. Tubulointerstistial fibrosis is regarded to be a final common pathway to end-stage kidney disease irrespectively of primary cause of kidney injury. This process is initiated at early stages of CKD and its degree is decisive for further prognosis of individual patient [10]. The presence of profibrogenic miRNAs in urine and serum has been considered to be a promising and non-invasive method of assessment of fibrosis in kidney diseases. In previous studies, it was demonstrated that miRNAs such as hsa-miR-21, hsa-miR-23a, hsa-miR-27, hsa-miR-34a-5p, hsa-miR-93, hsa-miR-132, hsa-miR-135, hsa-miR-155-5p, and hsa-miR-146 are highly expressed in renal fibrotic tissues and play pivotal role in kidney fibrogenesis. On the other hand hsa-miR-23b, hsa-miR-29, hsa-miR-30, hsa-miR-34a, hsa-miR-192, hsa-miR-200, and hsa-miR-433 are described downregulated and revealed an inhibitory effect on kidney fibrosis [11].

In our study, we found elevated expression of hsa-miR-29-5p, hsa-miR-21-5p, hsa-miR-196-5p in urine in analyzed CKD patients. Higher relative level of expression of hsa-miR-155-5p, hsa-miR-214-3p, hsa-miR-93-5p and hsa-miR-196a-5p was found in subgroup of CGN patients with DPE < 3.5 g. There are many studies of miR-21-5p role in inflammatory responses and kidney fibrosis and its association with TGFβ1/Smads family signaling pathway [12]. In our study, there was an increase of hsa-miR-21-5p urine expression. Increased urinary Mir-21 expression was also confirmed in patients with diabetic kidney disease (DKD), IgA nephropathy and polycystic kidney disease (PKD) [13, 14]. On the other hand, there are convincing data of hsa-miR-196a and hsa-miR-29a protective role against kidney fibrosis [15, 16]. The process of epithelial–mesenchymal transition (EMT) and renal fibrosis is also controlled by miR -200a and miR-141. We showed increased relative expression of hsa-miR-200a in serum in analyzed CKD group. These two miRNAs have also an important implication in renal fibrosis development as demonstrated with animal experimental models of acute kidney injury (AKI) and CKD also [17].

Another miRNAs evaluated in the study was hsa-miR-155-5p, which was higher in serum in subpopulation of CGN with eGFR > 60 ml/min and DPE > 3.5 g. Hsa–mir-155-5p seems to be an important part of tubulointerstitial damage. Hsa-miR-155-5p was shown to inhibit directly endothelial nitricoxide synthase (eNOS) production leading to increased oxidative stress. Inhibition of hsa-miR-155 may be a therapeutic target for improving endothelial dysfunction [10, 18].

In the experimental model of obstructive nephropathy inhibition of mir-29 by TGF-beta/Smad3 was related with enhanced renal fibrosis [19]. In our study, we found reduced relative expression level of hsa-miR-29 in serum of CKD patients but higher level in urine samples. This may be a sign of enhanced fibrosis intensity. There was also decreased level of expression of hsa-miR-21-5p, hsa-miR-196-5p, and hsa-miR-93-5p in serum also. These results may be interpreted as overactivation of fibrosis and augmented TGF–β activity may suppress above mentioned miRNAs. On the other hand the expression level of mir-29 was higher in serum in subpopulation of CGN patients with eGFR > 60/ml/min (Table 4). The expression of other profibrogenic miRNAs (hsa-miR-155-5p; hsa-miR-214-3p; hsa-miR-200a-5p, hsa-miR-196-5p) was also higher in serum of CNG patients with eGFR > 60 ml/min. It may indicate that changes of relative expression of some profibrogenic miRNAs can be detected at early CKD stages.

In conclusion, increased relative expression of some profibrogenic miRNAs in urine or serum of CKD patients with eGFR > 60 ml/min and DPE < 3.5 g may indicate higher degree of fibrosis at early CKD stages. The assessment of some miRNAs in urine or serum is considered to be a promising diagnostic method, especially in proteinuric kidney diseases.

The important limitation of the study was the fact that we were unable to assess serum or urine concentration of TGF-β, collagen or fibronectin which are strong markers of profibrotic processes. Moreover, we were unable to assess expression of abovementioned miRNAs in renal tissue obtained during kidney biopsy. This will be performed in further phase of the study. Further studies are required to assess the role of miRNAs as a diagnostics tool in kidney diseases.

## Data Availability

Not applicable.

## References

[1] Delanaye P, Glassock RJ, De Broe M (2017). Epidemiology of chronic kidney disease: think (at least) twice!. Clin Kidney J.

[2] Brück K, Stel VS, Gambaro G (2016). CKD prevalence varies across the European general population. J Am Soc Nephrol.

[3] Nangaku M (2004). Mechanisms of tubulointerstitial injury in the kidney: final common pathways to end-stage renal failure. Intern Med.

[4] Rodriguez-Iturbe B, Johnson RJ, Acosta J (2005). Tubulointerstitial damage and progression of renal failure. Kidney Int Suppl.

[5] Zhao H, Ma SX, Shang YQ, Zhang HQ, Su W (2019). MicroRNAs in chronic kidney disease. Clin Chim Acta.

[6] Metzinger-Le Meuth V, Fourdinier O, Charnaux N (2019). The expanding roles of microRNAs in kidney pathophysiology. Nephrol Dial Transplant.

[7] Wang GK, Zhu JQ, Zhang JT (2010). Circulating microRNA: a novel potential biomarker for early diagnosis of acute myocardial infarction in humans. Eur Heart J.

[8] Gniewkiewicz M, Paszkowska I, Gozdowska J (2020). Urinary microRNA-21-5p as potential biomarker of interstitial fibrosis and tubular atrophy (IFTA) in kidney transplant recipients. Diagnostics (Basel).

[9] Cheng Y, Wang D, Wang F (2020). Endogenous miR-204 protects the kidney against chronic injury in hypertension and diabetes. J Am Soc Nephrol.

[10] Chung AC, Lan HY (2015). MicroRNAs in renal fibrosis. Front Physiol.

[11] Zhang W, Li X, Tang Y (2020). miR-155–5p implicates in the pathogenesis of renal fibrosis via targeting SOCS1 and SOCS6. Oxid Med Cell Longev.

[12] Loboda A, Sobczak M, Jozkowicz A (2016). TGF-β1/smads and miR-21 in renal fibrosis and inflammation. Mediators Inflamm.

[13] Wang G, Kwan BC-H, Lai FM (2012). Urinary miR-21, miR-29 and miR-93: novel biomarkers of fibrosis. Am J Nephrol.

[14] Lange T, Artelt N, Kindt F (2019). MiR-21 is up-regulated in urinary exosomes of chronic kidney disease patients and after glomerular injury. J Cell Mol Med.

[15] Zhang C, Liang S, Cheng S (2018). Urinary miR-196a predicts disease progression in patients with chronic kidney disease. J Transl Med.

[16] Wang H, Wang B, Zhang A (2019). Exosome-mediated miR-29 transfer reduces muscle atrophy and kidney fibrosis in mice. Mol Ther.

[17] Aguado-Fraile E, Ramos E, Conde E (2013). MicroRNAs in the kidney: novel biomarkers of acute kidney injury. Nefrologia.

[18] Shaffi SK, Galas D, Etheridge A (2018). Role of microRNAs in renal parenchymal diseases—a new dimension. Int J Mol Sci.

[19] Qin W, Chung ACK, Huang XR (2011). TGF-β/smad3 signaling promotes renal fibrosis by inhibiting miR-29. J Am Soc Nephrol.

